# Hybrid Vesicle Stability under Sterilisation and Preservation Processes Used in the Manufacture of Medicinal Formulations

**DOI:** 10.3390/polym12040914

**Published:** 2020-04-15

**Authors:** Rashmi Seneviratne, Lars J. C. Jeuken, Michael Rappolt, Paul A. Beales

**Affiliations:** 1 School of Chemistry, Astbury Centre for Structural Molecular Biology and Bragg Centre for Materials Research, University of Leeds, Leeds LS2 9JT, UK; cm12rhs@leeds.ac.uk; 2 School of Biomedical Sciences, Astbury Centre for Structural Molecular Biology and Bragg Centre for Materials Research, University of Leeds, Leeds LS2 9JT, UK; L.J.C.Jeuken@leeds.ac.uk; 3 School of Food Science and Nutrition and Bragg Centre for Materials Research, University of Leeds, Leeds LS2 9JT, UK; M.Rappolt@leeds.ac.uk

**Keywords:** liposomes, polymersomes, filtration, freeze-thaw, membrane mechanics, encapsulation stability

## Abstract

Sterilisation and preservation of vesicle formulations are important considerations for their viable manufacture for industry applications, particular those intended for medicinal use. Here, we undertake an initial investigation of the stability of hybrid lipid-block copolymer vesicles to common sterilisation and preservation processes, with particular interest in how the block copolymer component might tune vesicle stability. We investigate two sizes of polybutadiene-*block*-poly(ethylene oxide) polymers (PBd_12_-PEO_11_ and PBd_22_-PEO_14_) mixed with the phospholipid 1-palmitoyl-2-oleoyl-sn-glycero-3-phosphocholine (POPC) considering the encapsulation stability of a fluorescent cargo and the colloidal stability of vesicle size distributions. We find that autoclaving and lyophilisation cause complete loss of encapsulation stability under the conditions studied here. Filtering through 200 nm pores appears to be viable for sterilisation for all vesicle compositions with comparatively low release of encapsulated cargo, even for vesicle size distributions which extend beyond the 200 nm filter pore size. Freeze-thaw of vesicles also shows promise for the preservation of hybrid vesicles with high block copolymer content. We discuss the process stability of hybrid vesicles in terms of the complex mechanical interplay between bending resistance, stretching elasticity and lysis strain of these membranes and propose strategies for future work to further enhance the process stability of these vesicle formulations.

## 1. Introduction

Hybrid vesicles aim to combine the properties of biomimetic liposomes and synthetic polymersomes into composite membrane-bound capsules with broader tuneability of material properties [1,2,3,4,5,6,7,8,9,10]. This expanded material parameter space is anticipated to be beneficial in the development of hollow vesicle structures for technological applications such as sensors, biotechnology, nanoreactors, synthetic biology and formulated products, including those intended for medicinal purposes [11,12,13,14,15,16,17,18,19,20,21].

Individually, liposomes and polymersomes offer different advantages [22]. Liposomes, composed of natural lipids, form bilayer membranes that closely mimic the structural matrix of native biomembranes. This makes them highly biocompatible and provides a native-like environment if integral membrane proteins are desired to add functionality to the membrane [23,24,25,26,27]. However, liposomes can have inherent instabilities. Their membranes are highly flexible under bending deformations, but weak under stretching deformations, with a lysis strain of less than 5% [28,29]. The labile fluidity of the membrane can lead to transient membrane defects that frustrates long term encapsulation stability and lipid peroxidation can cause chemical instabilities in these structures. 

Polymersomes, due to their synthetic nature, are often less biocompatible than their lipid counterparts but offer greater mechanical stability and a broader chemical parameter space [30]. Amphiphilic copolymers that form vesicles can have several different architectures, the most common being AB diblock (A = hydrophilic, B = hydrophobic) [6], ABA [31] and ABC tri-block polymers [32] where A and C are chemically different hydrophilic blocks and finally graft copolymers [33]. Formation of polymersomes depends on the amphiphilic co-polymers molecular weight, polydispersity and hydrophilic/hydrophobic block lengths ratio, which is ideally less than 1:3 to form vesicular structures [34]. Their membranes are often thicker than liposome membranes, which provides greater bending resistance and their enhanced elasticity under stretching makes them tough and durable [22]. The polymer chemistry can be designed to minimise chemical instability but also to incorporate novel functionality. In blending liposomes and polymersomes to create hybrid vesicles, the ambition is to combine advantageous properties of these materials while off-setting their weaknesses [35].

For medicinal applications in particular, stringent regulatory requirements exist for the sterilisation and preservation during the transportation and storage of these formulations [36]. Vesicles need to exhibit good stability under appropriate processing conditions that maintain the encapsulation of active compounds and the colloidal stability of vesicle structures.

**Figure 1:**
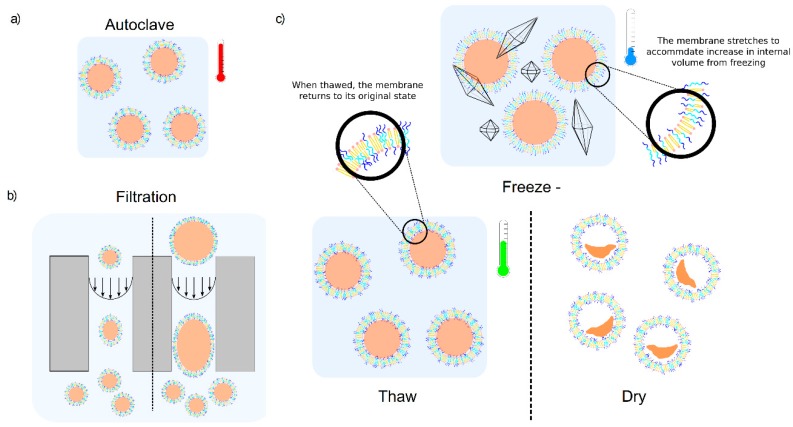
Schematic of sterilisation and preservation processes applied to vesicle formulations. (**a**) Autoclaving; (**b**) filtration; (**c**) freeze-thaw and lyophilisation.

Sterilisation is required to make the product safe for public use. It is the process by which all forms of life are destroyed, removed or permanently inactivated. Instead of an absolute measure of sterilisation, pharmaceutical industries use processes that reduce the probability of the survival of undesired organisms to a negligible level. Currently, pharmaceutical companies use thermal, filtration and irradiation techniques to sterilise their products [36,37]. Thermal sterilisation (Figure 1a), by using an autoclave, is the most common and the most reliable technique as it achieves destruction of microorganisms by irreversible denaturation of enzymes [36] and it allows the sterilisation of larger objects more easily than filtration [38].

Filtration through a maximum of a 0.22 µm membrane is usually used for sterilisation of thermally labile solutions, while sterilisation of active ingredients or medical devices can be achieved by irradiation [37]. Filtration exerts fluid shear stresses on vesicles as they pass down channels in the filter membrane (Figure 1b). While vesicles smaller than the filter pore size might fit within the channel without needing to deform, under flow, the Poiseuille velocity flow field in the channel indicates the fluid is moving much faster in the centre of the pore channel than at the edges. This will deform even small vesicles and, depending on the magnitude of these stresses, could induce transient pore formation in the membrane, compromising encapsulation stability, or even causing break-up of the vesicles. For larger vesicles, greater in size than the pore diameter, these vesicles must also deform at the entrance to the channel and will experience greater shear stresses that increases the probability that these vesicles might break-up into smaller structures.

Stability during the storage and transportation of vesicle formulations can also be a challenge. This may be in the transport between primary and secondary manufacturing sites or between the manufacturer and end-user. Once reaching the end-user, formulations may be stored for some time before their final use. Cold chain transportation is frequently used for medicinal products to maintain the stability and activity of their ingredients. Cryogenic conditions are frequently used for preservation during the transportation and storage of delicate biologics and labile formulations.

Liquid formulations are often frozen for preservation (Figure 1c). However, storing frozen samples for an extended period can cause ice crystal formation that could cause membrane damage for vesicles during freezing and thawing [39]. Besides potential damage from ice crystals, vesicles will become stressed by the expansion of water inside their lumen upon freezing. The volume of water expands by ~9% upon freezing, which will exert a ~6% areal strain on the confining membrane, which exceeds the lysis strain for lipid membranes. Indeed, freeze-thaw cycles are used to intentionally rupture liposomes during the passive loading of compounds from the bulk medium. Formulations may experience several freeze-thaw processes during manufacture, transport and storage, whether deliberate or unintentional. Fluctuations in temperature during transport from improper storage and shipping can cause some samples to reach their destination past their shelf life. This makes some drug formulations ineffective or even harmful [40,41].

Lyophilisation (freeze-drying) can also be used to maintain the stability of lipids during transportation and storage. During the freeze-drying process the product is frozen, thus immobilising the sample and allowing it to retain its original form, and then the water is removed by sublimation (Figure 1c), preventing microbial growth [42]. For lipids, the absence of water would minimise the rate of hydrolysis during storage [43]. However lyophilisation of liposome formulations is challenging to maintain encapsulation and structural stability. Additives are required for liposome cryopreservation, yet their efficacy can be variable and inconsistent [44].

Here, we investigate hybrid vesicles composed of a phospholipid 1-palmitoyl-2-oleoyl-sn-glycero-3-phosphocholine (POPC) and one of two amphiphilic polybutadiene-block-polyethylene oxide (PBd-*b*-PEO) diblock copolymers of different molecular weights under conditions designed to mimic industrial sterilisation and storage/transportation processes. We explore the full hybrid compositional space from pure lipid to pure polymer vesicles. Sterilisation is mimicked through autoclaving or multiple filtration passes through a filter with a 0.2 μm pore size. The storage/transportation of samples is emulated through either lyophilisation and rehydration, or multiple freeze-thaw-vortex (FTV) cycles. This preliminary study of hybrid vesicle stability under these conditions is designed as a stringent test of their stability that will highlight advantages that can be gained through the composition of the hybrid formulation and to uncover aspects of these formulations that will require optimisation for future commercial translation. As such, we investigate the hybrid vesicle in a physiological saline buffer without additional additives for enhanced stability, such as potential cryo-preservatives.

## 2. Materials and Methods

### 2.1. Materials

The di-block copolymers poly(1,2-butadiene)-*block*-(polyethylene oxide) (PBd-*b*-PEO) with total molecular weights of 1800 gmol^−1^ and 1150 gmol^−1^ were purchased from Polymer Source, Inc. (Montreal, Canada). PBd_22_-PEO_14_ (PDI 1.01) has a hydrophobic PBd block of 1200 gmol^−1^ (>85% 1,2 addition) and a hydrophilic PEO block of 600 gmol^−1^ while polybutadiene-*block*-poly(ethylene oxide) (PBd_12_-PEO_11_) (PDI 1.09) purchased from Polymer Source (Dorval, Montreal, Canada) has a hydrophobic PBd block of 650 gmol^−1^ (85% 1,2 addition) and a hydrophilic PEO block of 500 gmol^−1^.

The lipid 1-palmitoyl-2-oleoyl-sn-glycero-3-phosphocholine (POPC) was purchased from Avanti Polar Lipids (Alabaster, AL, USA). 5(6)-carboxyfluorescein (CF) and other reagents were purchased from Sigma Aldrich (St. Louis, MO, USA).

### 2.2. Vesicle Preparation

Large unilamellar vesicles (LUVs) were prepared by the thin film rehydration and extrusion method. To generate different hybrid vesicle compositions, relative volumes of POPC (32 mM) and PBd-PEO (6.57 mM) in chloroform were measured using a Hamilton syringe into a glass vial. The solutions were dried in a vacuum desiccator to give a lipid/polymer film and then rehydrated with 1.0 mL of aqueous solution of 60 mM CF with 40 mM 4-(2-hydroxyethyl)-1-piperazineethanesulfonic acid (HEPES) and 20 mM sodium chloride, adjusted the pH to 7.4 by dropwise addition of sodium hydroxide. The films were incubated at 50 °C for 5 min and vortexed for 1 min. The suspensions were then frozen in liquid nitrogen, thawed in a 60 °C water bath and vortexed for 10 s. This cycle was repeated 5 times. Suspensions were extruded 11 times though a 100 or 400 nm pore size polycarbonate membrane filters using a LiposoFast Basic Extruder. The nanovesicle samples were run on a Sephadex G50 column under gravity using 40 mM HEPES and 20 mM sodium chloride buffered to pH 7.4 as the mobile phase to remove unencapsulated CF dye. The resulting 3 mL fractions were characterised using dynamic light scattering (DLS) for particle size distribution to confirm the presence of vesicles. The hybrid vesicles are then further analysed for stability under sterilisation and preservation processes.

### 2.3. CF Release Assay

The 100 nm sized hybrid vesicle samples were split into 4 fractions of 500 µL (~2 mM). Fraction 1 was thermally sterilised using an autoclave at 121 °C for 15 min. Fraction 2 was lyophilised using a VirTis Benchtop Pro Lyophiliser (Wolf Laboratories Ltd., York, UK) for 24 h after freezing the sample in nitrogen. Fraction 3 underwent 5 filtration cycles through a 13 mm PTFE 200 nm syringe filter device (Fisher Scientific Ltd., Hampton, NH, USA) with polypropylene housing. Fraction 4 was frozen in liquid nitrogen, thawed in a water bath at 60 °C and vortexed for 3 s. The samples undergo 4 of these freeze-thaw-vortex (FTV) cycles.

The 400 nm sized vesicles were filtered through a 13 mm PTFE 200 nm syringe filter device (Fisher Scientific Ltd., Hampton, NH, USA) with a polypropylene housing 5 times.

CF is self-quenching at high concentrations (>40 mM) [45,46], so the emission intensity at this concentration was often very low. The CF was encapsulated at 60 mM, so the control samples would have a low emission intensity. When the vesicles release the encapsulated CF, the CF was diluted by the external buffer and the fluorescence intensity increased. 

To measure the CF release, 0.5 mL fractions were diluted to 2 mL (~18 µM) and the fluorescence emission at 519 nm of CF-encapsulated vesicles was measured with excitation set to 492 nm using a Horiba Scientific FluoroMax Plus (Horiba Ltd., Kyoto, Japan). Measurements were made on initial vesicle fractions and between every processing cycle for each fraction. The initial vesicle preparations were ruptured with 50 µL of 10% (*w*/*v*) Triton X-100 (end concentration 0.91% (*w*/*v*)) (Scientific Laboratory Supplies Ltd., Nottingham, UK) to completely destabilise the vesicles and release the encapsulated CF before a final fluorescence emission at 519 nm was measured.

The CF % release was calculated by

(1) $$\%\;CF\;release=\frac{I_{i}-I_{0}}{I_{t}-I_{0}}\times 100\%$$

where $I_{0}$ is the initial intensity of the sample, $I_{i}$ is the intensity of the sample after each processing cycle (from autoclaving, lyophilisation, FTV or filtering), and $I_{t}$ is the intensity after the initial sample is burst with Triton X-100.

### 2.4. Dynamic Light Scattering (DLS)

After separating hybrid vesicles with encapsulated CF from excess CF, the vesicles were characterised using a Malvern Zetasizer Nano ZSP (Malvern Panalytical Ltd., Malvern, UK) with scattering angle 173° to determine their size. Each hybrid vesicle sample (0.5 mM) was measured at 25 °C by the DLS and the results from 3 independent repeats were averaged. Size distributions were also measured after the processing steps described above to assess the colloidal stability of these vesicle formulations under these conditions.

### 2.5. Cryo-Electron Microscopy (cryo-EM)

Vesicles of 50 mol% PBd_22_-PEO_14_ at 100 and 400 nm were imaged after initial preparation and after the final freeze-thaw-vortex and filtration cycles respectively. For 100 nm vesicles, 400 mesh 2/2 µm Cu Quantifoil grids were used, while for 400 nm vesicles, 400 mesh Cu Lacey grids were used. All the carbon-coated grids were glow discharged for 33 s at 10 mA to render the surface hydrophilic. Samples (3 µL, ~2 mM) were placed directly onto the grid with a hold time of 40 s, using a FEI Vitrobot mkIV, using a blotting time of 6 s and a blot force of 6. The samples were kept in closed cryo-pucks under liquid nitrogen until required.

To image the girds, an FEI Titan KRIOS microscope (Thermofisher, Waltham, MA, USA) with an accelerating voltage of 300 kV was used with a defocussing of 3 µm at a magnification of ×130,000 and ×26,000. The resolution for these images was 0.11 and 0.55 nm/pixel, respectively.

Quantitative analysis of the images required taking diameter measurements and observations of all the vesicles in the grid holes of the carbon-coated copper grid using ImageJ. Histograms were made to document the size distribution observed in the grid holes, and notes were made on the ratio of multilamellar vesicles (MLVs) to unilamellar vesicles (ULVs). 

## 3. Results

We investigated the stability of hybrid vesicles under conditions designed to mimic industrial sterilisation and preservation processes. Our hybrid vesicles were composed of the lipid POPC blended with one of two PBd-*b*-PEO diblock copolymers of different length: PBd_22_-PEO_14_ or PBd_12_-PEO_11_. We explored the full compositional parameter space from pure lipid to pure polymer vesicle compositions. Hybrid vesicles were formed by the thin film rehydration and extrusion method. The vesicles were loaded with the hydrophilic fluorophore CF as a model encapsulated compound. We studied the encapsulation stability through release of CF from vesicles by fluorescence spectroscopy and the colloidal stability of the vesicle formulations from their hydrodynamic size distributions obtained by DLS.

### 3.1. Autoclaving and Lyophilisation

**Figure 2:**
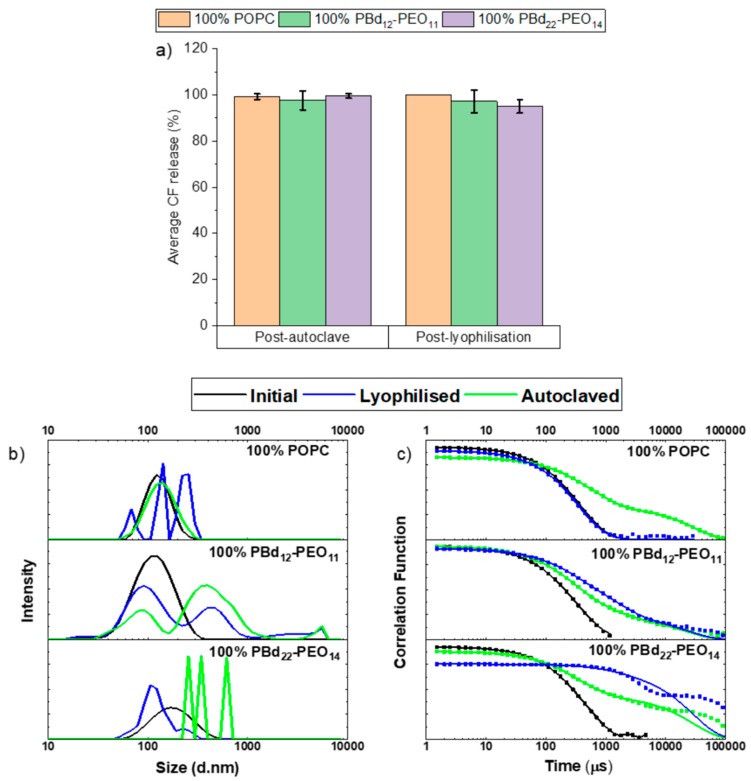
Release of encapsulated 5(6)-carboxyfluorescein (CF) and size distributions from vesicles following autoclaving or lyophilisation. (**a**) Vesicle compositions 100% 1-palmitoyl-2-oleoyl-sn-glycero-3-phosphocholine (POPC), 100% polybutadiene-*block*-poly(ethylene oxide) (PBd_12_-PEO_11_) and 100% PBd_22_-PEO_14_ were investigated following autoclaving or lyophilisation. Each measurement was performed in triplicate and the errors data points represent mean ± s.d. The dynamic light scattering (DLS) distributions of hydrodynamic diameters from (**b**) constrained regularisation method for inverting data (CONTIN) fits size distributions and (**c**) the fitted autocorrelation functions. The size distributions shown represent the average distribution from three independent repeats.

Autoclaving and lyophilisation of hybrid vesicles would be the preferred choices for sterilisation and preservation, if these formulations can be made to be stable under these conditions. Unfortunately, both processes proved to be too destructive, causing vesicles to become unstable and release their entire contents. Only vesicles composed of the pure components were investigated: 100% POPC, 100% PBd_22_-PEO_14_ and 100% PBd_12_-PEO_11_. As 100% CF release was observed in all cases (Figure 2a), it was considered extremely unlikely that hybrid blends would perform any better.

The colloidal stability of the vesicles under these conditions was similarly poor. DLS size distributions were collected before and after autoclaving and lyophilisation (Figure 2b). The fitting software returned errors for all post-process samples except for autoclaving of 100% liposomes. The poor fits to the auto-correlation function in these cases can be seen in Figure 2c. The delayed exponential decay shown in the auto-correlation functions is interpreted as aggregation and/or structural instabilities of the vesicles during autoclaving or lyophilisation. The fact that the autocorrelation function does not reach the baseline in some cases indicates the presence of larger multimicron structures that are likely to undergo sedimentation.

Since these vesicles are unstable under autoclaving and lyophilisation, we further investigate other potential methods of sterilisation and preservation. However, we do not conclude that these techniques are fundamentally hopeless for use with hybrid vesicles: further development of the vesicle formulations would be required; for example, additional additives to the solution environment that help protect the integrity of the vesicles undergoing these processes.

### 3.2. Filtration

Sterilisation can be achieved by filtrations through a membrane with a maximum pore size of 0.22 µm. Here, we use a 0.2 µm pore size membrane to filter hybrid vesicles for between one and five cycles. Initially, we investigated hybrid vesicles between 0 and 100 mol% block copolymer content in 25 mol% increments for both PBd-*b*-PEO polymers. These vesicles were formed by extrusion through a 100 nm pore size membrane and encapsulated the CF dye. 

**Figure 3:**
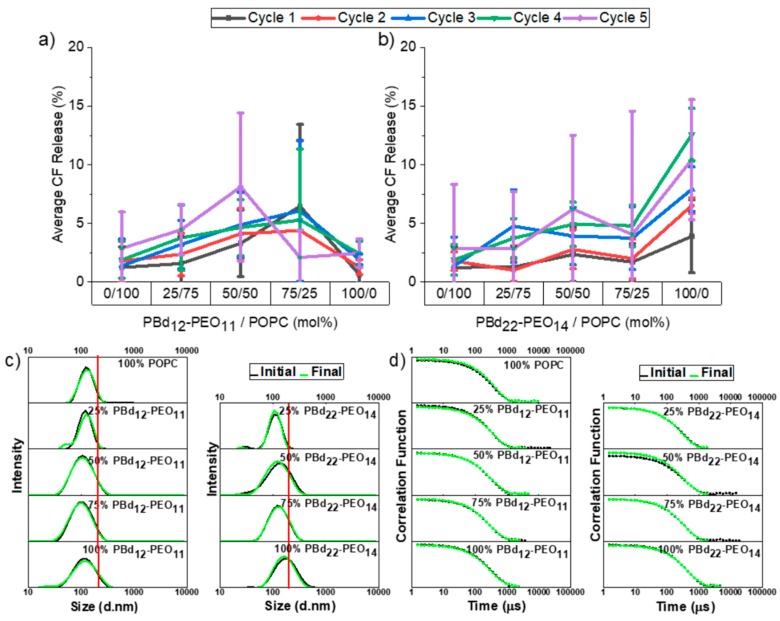
Release of encapsulated CF following filtration and size distributions before and after five filtration cycles. CF release% is plotted against membrane composition for hybrid vesicles composed of POPC and (**a**) PBd_12_-PEO_11_, or (**b**) PBd_22_-PEO_14_. Data are shown for between one and five filtration cycles through a 0.2 µm pore size filter membrane. Each measurement was performed in triplicate and the errors data points represent mean ± s.d. The DLS distributions of hydrodynamic diameters of PBd_12_-PEO_11_ or PBd_22_-PEO_14_ hybrid vesicles before and after five filtration cycles: (**c**) CONTIN fit size distributions; (**d**) the fitted autocorrelation functions. The size distributions shown represent the average distribution from three independent repeats. The line at 0.2 μm denotes the filtration pore size.

All vesicle compositions only exhibited minimal contents release across five filtration cycles, with the average contents release reaching at most 10%. Figure 3a shows filtration-induced contents release from hybrid vesicles containing the shorter PBd_12_-PEO_11_ block copolymer. The unitary POPC and PBd_12_-PEO_11_ vesicles exhibit very low (<5%) contents release across five filtration cycles and no vesicle composition exceeds 10% average release. Analysis of variance shows that there is no statistically significant (p < 0.05) difference between the different vesicle compositions studied.

Perhaps surprisingly, the largest contents release occurred in vesicles composed of the larger PBd_22_-PEO_14_ block copolymer (Figure 3b), where a priori expectations might be that these would be the most mechanically stable vesicles. The only statistically significant difference in contents release is for pure PBd_22_-PEO_14_ polymersomes, which is significant when compared to all other membrane compositions, where the extent of release marginally exceeds 10% for the fourth and fifth filtration cycle. However, this contents release is still relatively low, even after several filtration cycles.

Hybrid vesicles (100 nm extrusion pore size) also demonstrate colloidal stability following multiple filtration cycles (Figure 3c,d and Tables S1–S4). Vesicle size distributions obtained by DLS before and after five filtration cycles were comparable for all hybrid vesicle compositions and both polymer sizes. No evidence of break-up or aggregation of vesicles was observed. The red line on these graphs denotes the filter pore size of 200 nm. Vesicles with higher polymer content exhibit a tail in their size distribution which stretches beyond the filtration pore size. While these vesicles can pass the filter without notable changes in the particle size distribution, these also largely correlate with the vesicle compositions that exhibit the highest contents release, suggesting that the probability of transient pores forming in the vesicle membrane increases when vesicles are larger than the pore size and must significantly deform during the transit of the pore channel. This is particularly notable for the size distribution of 100 mol% PBd_22_-PEO_14_ vesicles, where the highest contents release was measured in Figure 3b.

Given that most of the 100 nm extruded vesicles are smaller than the 200 nm filter size, we presented a more stringent test of hybrid vesicle stability under filtration by using 400 nm extruded vesicles. In this series of experiments, we investigated the pure lipid and polymer vesicles as well as the 50 mol% hybrid compositions by both polymer sizes.

**Figure 4:**
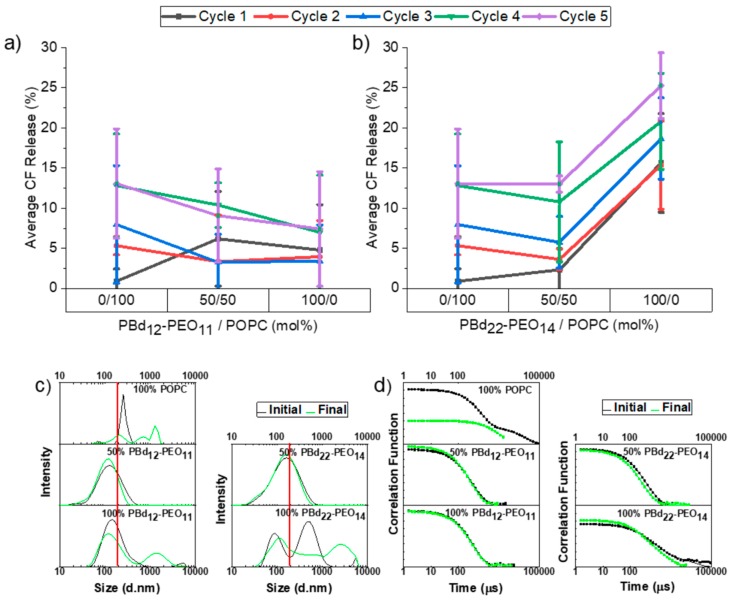
Release of encapsulated CF from 400 nm hybrid vesicles following filtration and size distributions before and after five filtration cycles. CF release% is plotted against membrane composition for hybrid vesicles composed of POPC and (**a**) PBd_12_-PEO_11_, or (**b**) PBd_22_-PEO_14_. Data are shown for between one and five filtration cycles through a 0.2 µm pore size filter membrane. Each measurement was performed in triplicate and the errors data points represent mean ± s.d. The DLS distributions of hydrodynamic diameters of PBd_12_-PEO_11_ or PBd_22_-PEO_14_ hybrid vesicles before and after five filtration cycles: (**c**) CONTIN fit size distributions; (**d**) the fitted autocorrelation functions. The size distributions shown represent the average distribution from three independent repeats. The line at 0.2 μm denotes the filtration pore size.

Contents release from 400 nm extruded vesicles was significantly greater than previously observed for 100 nm extruded vesicles (Figure 4). While the shorter PBd_12_-PEO_11_ polymer appears to provide a mildly protective effect in hybrid vesicles with a gentle reduction in CF release observed with increasing polymer composition, these differences are not statistically significant (Figure 4a). The only statistically significant difference in contents release is again observed for pure PBd_22_-PEO_14_ polymer vesicles, which show reduced encapsulation stability compared to the POPC liposomes and 50 mol% hybrid vesicles (Figure 4b). Despite this, overall contents release after five filtration cycles is small, being less than 15% for all compositions except the 100 mol% PBd_22_-PEO_14_ vesicles, where contents release exceeds 25% after five filter passes.

The filtration of PBd_22_-PEO_14_ vesicles was more difficult than other vesicle compositions, requiring much higher pressures to be applied to force the vesicle sample through the filter. This indicates that these vesicles are the most rigid due to the high bending stiffness of the thicker polymer membranes. The higher pressures that need to be applied during filtration are therefore the likely cause of the enhanced contents release from these vesicles. On the other hand, the shorter polymer has lower bending resistance, allowing these vesicles to more easily pass through the filter and may impart some enhanced elasticity to hybrid vesicles, giving a mild protective effect to hybrid vesicles under filtration.

All vesicles were much smaller in size than the 400 nm pore size of the extrusion membrane for vesicle preparation (Figure 4c,d and Tables S5–S8). However, a significant proportion of vesicles are larger than the 200 nm filter size, meaning that they would need to significantly deform or break-up to pass through the filter. The initial 100% PBd_22_-PEO_14_ vesicle size distribution also showed a bimodal distribution with peaks at 108 and 519 nm. Following five filter passes, all pure component vesicles, whether lipid or polymer, showed multimodal size distributions with larger structures present in solution that are indicative of aggregation and loss of colloidal stability in these samples.

Interestingly, both 50% hybrid vesicle formulations fared best under filtration with comparable monomodal size distributions before and after filtration. This enhanced colloidal stability might be due to the synergistically enhanced properties of the blended lipid-polymer hybrid membranes. However, it is also plausible that this is simply due to the smaller initial size distribution of these hybrid vesicles (145 nm for 50/50 mol% POPC/PBd_12_-PEO_11_ hybrid vesicles and 182 nm for 50/50 mol% POPC/PBd_22_-PEO_14_ hybrid vesicles). We previously observed good colloidal stability for 100 nm extrusion membrane vesicle formulations, therefore this latter explanation seems most likely. This smaller initial size distribution of 50 mol% hybrid vesicles might be due to a preferred curvature of the hybrid vesicle membrane that limits the maximum size of vesicles that form at this composition.

**Figure 5:**
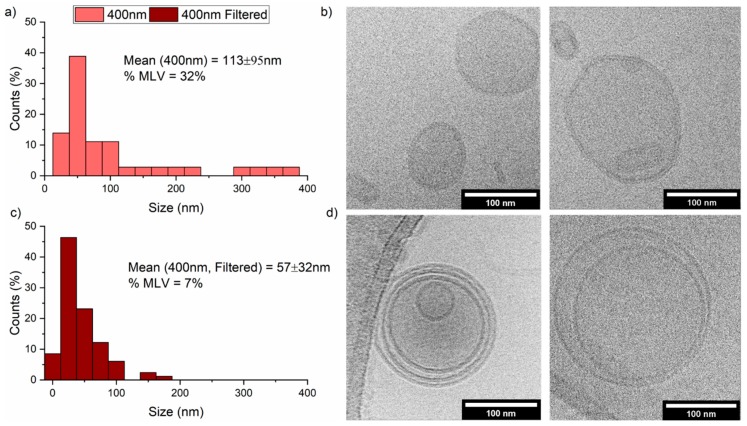
Cryo-transmission electron microscopy (Cryo-TEM) of 50 mol% PBd_22_-PEO_14_ hybrid vesicle (400 nm extrusion membrane) before and after five filtration cycles. (**a**) Histogram of initial vesicle sizes from cryo-TEM images; (**b**) representation images of vesicles before filtration; (**c**) histogram of vesicle sizes after filtration from cryo-TEM images; (**d**) representation images of vesicles after filtration. Scale bars represent 100 nm.

We further investigated the 400 nm 50/50 mol% POPC/PBd_22_-PEO_14_ hybrid vesicles before and after filtration by cryo-electron microscopy (cryo-EM) (Figure 5). For cryo-EM, an aqueous sample is placed on a carbon-coated copper grid which is plunged in liquid ethane that vitrifies the water into a glass-like state to enable the sample to be seen [47,48]. Ice thickness can vary depending on the dimensions of the particle but usually ranges from a few nanometres to a hundred nanometres [48]. The thicker the ice (>100 nm), the worse the resolution [49]. On the other hand, if the ice is too thin, then either the sample is pushed towards the edge of the grid holes, or the sample can have a high affinity for the carbon support, leaving the grid hole empty of sample and causing particle aggregation [47]. Variations in ice thickness can also sort the vesicles by size, concentrating the larger vesicles in thicker ice [50]. Lacey carbon-coated grids have a higher percentage of open area which allows larger particles to protrude over the edges of the grid holes, and therefore be seen at a higher resolution [51].

Initial 50 mol% PBd_22_-PEO_14_ hybrid vesicles were found to have an average diameter of 113 ± 95 nm and 32% of vesicles were observed to be multilamellar. This compares favourably with the previous DLS analysis where these vesicles have a z-average of 124 nm (PDI 0.279). However, following filtration, the cryo-EM analysis gave an average vesicle size of 57 ± 32 nm with 7% of vesicles found to be multilamellar. This differs from our DLS analysis, which showed a post-filtration z-average of 120 nm (PDI 0.269). The reduction in average size observed in cryo-EM can be seen from the histograms to be due to the removal of larger >200 nm vesicles from the post-filtered samples, likely due to vesicle break-up. Vesicle break-up during filtration would also explain the lower proportion of multilamellar vesicles that we observe. While larger vesicles can often be excluded from the thin ice in the holes of the EM grid, artificially leading to smaller size distributions by cryo-EM compared to DLS, the comparison between the pre- and post- filtered hybrid vesicles by cryo-EM would appear to be significant. The break-up of some larger and multilamellar vesicles during filtration would likely account for the ~15% leakage of vesicle contents observed in Figure 4.

### 3.3. Freeze-Thaw-Vortex

An alternative preservation process to lyophilisation is to store vesicle samples frozen from the liquid state without sublimation of excess water. Therefore, we investigate the stability of vesicles to up to four freeze-thaw-vortex (FTV) cycles. The vortexing step following the thawing of frozen samples is included to ensure the vesicles are fully resuspended in the liquid state.

**Figure 6:**
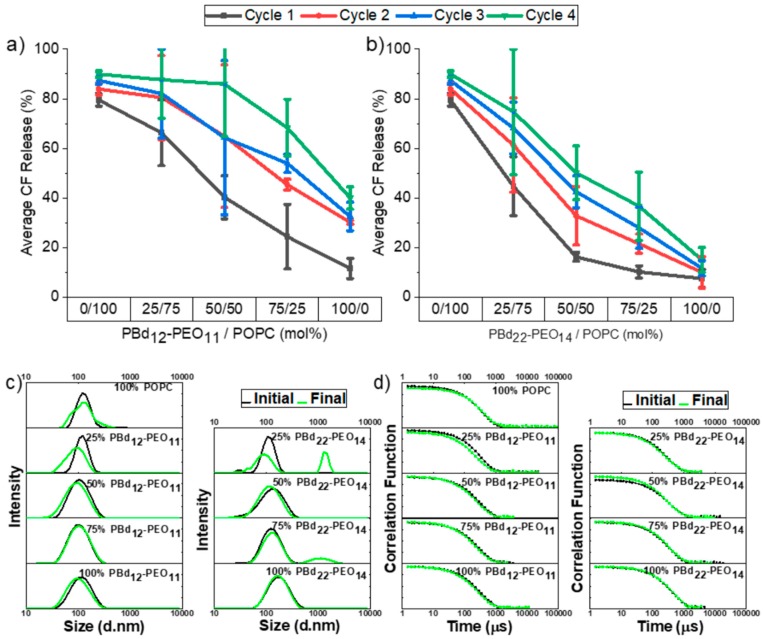
Release of encapsulated CF from 100 nm hybrid vesicles following freeze-thaw-vortex (FTV) cycles and size distributions before and after four FTV cycles. CF release% is plotted against membrane composition for hybrid vesicles composed of POPC and (**a**) PBd_12_-PEO_11_, or (**b**) PBd_22_-PEO_14_. Data are shown for between one and four FTV cycles. Each measurement was performed in triplicate and the errors data points represent mean ± s.d. The DLS distributions of hydrodynamic diameters of PBd_12_-PEO_11_ or PBd_22_-PEO_14_ hybrid vesicles before and after four FTV cycles: (**c**) CONTIN fit size distributions; (**d**) the fitted autocorrelation functions. The size distributions shown represent the average distribution from three independent repeats.

Liposome encapsulation was observed to be highly unstable even after a single FTV cycle (Figure 6). This is unsurprising as freeze-thaw cycles are frequently used for passive loading of cargo in liposome preparation protocols, as this renders the membrane temporarily permeable. Conversely, 100% polymer vesicles exhibit much greater encapsulation stability across four FTV cycles with hybrid formulations showing increasing stability with increasing block copolymer composition in the membranes. For all vesicle compositions, CF release increased after each FTV cycle.

Vesicles containing the larger PBd_22_-PEO_14_ polymer were also observed to have more stable CF encapsulation than those formulated with the PBd_12_-PEO_11_ polymer. We interpret this as the thicker membranes formed from the larger block copolymer having greater elasticity, permitting the membrane to stretch without rupture during the volume expansion of freezing. Furthermore, the thicker, more elastic membranes of these vesicles enhances protection from damage that might be caused by the local growth of ice crystals in the sample.

FTV cycles had the most impact on the colloidal stability of lipid-rich vesicles. We measured the vesicle size distributions by DLS before and after four FTV cycles (Figure 6c,d and Tables S9–S12). While the post-FTV size distributions are mostly monomodal (with the exception of 25 mol% and 75 mol% PBd_22_-PEO_14_ hybrid vesicles), the size distributions for 100% POPC and 25 mol% polymer hybrid vesicles significantly broaden following four FTV cycles. In contrast, the size distributions of hybrid vesicles with ≥50 mol% polymer content are broadly similar before and after the FTV cycles. 

**Figure 7:**
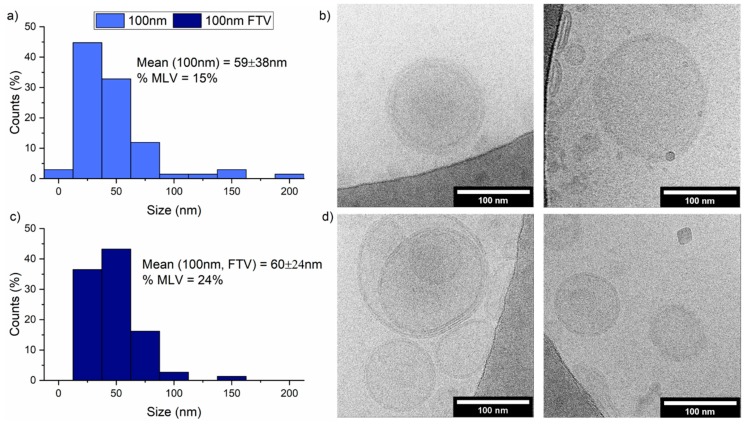
Cryo-TEM of 100 nm 50 mol% PBd_22_-PEO_14_ hybrid vesicles before and after four FTV cycles. (**a**) Histogram of initial vesicle sizes from cryo-TEM images; (**b**) representation images of vesicles before FTV; (**c**) histogram of vesicle sizes after FTV from cryo-TEM images; (**d**) representation images of vesicles after FTV. Scale bars represent 100 nm.

We further investigate the effect of freeze-thaw action on the size and morphology of 50 mol% PBd_22_-PEO_14_ hybrid vesicles by cryo-EM. Quantitative analysis of the images shown in Figure 7 indicate that the vesicles have a similar size before and after the FTV cycles (59 ± 38 vs. 60 ± 24 nm) and a slightly increased prevalence of multilamellar vesicles following FTV cycles (15% vs. 24%). However the cryo-EM grid preparation appears to favour smaller vesicles being in the grid holes, while the majority of the larger vesicles prefers to sit on the carbon-coated support as there is a large difference in size between the DLS and cryo-EM size distributions. The DLS analysis in Figure 6c reports z-average sizes of 108 nm (PDI 0.213) and 101 nm (PDI 0.191) before and after four FTV cycles, respectively.

## 4. Discussion

For vesicles composed of the pure lipid or block copolymer components, we find that sterilisation using an autoclave results in a loss of colloidal stability and the release of the encapsulated CF. Autoclaving involves exposure to high temperature, which is thought to be detrimental to lipid vesicles by causing aggregation [38,52]. The aggregation after heat sterilisation has been suggested to lead to instability in lipid vesicles by electrolytes, causing dehydration of the hydrophilic moiety [52]. However, in a previous study, it was found that egg or saturated lipid vesicles without encapsulated agents could be sterilised by autoclaving. When calcein or doxorubicin were encapsulated, however, the leakage was pronounced [38].

It was expected that the polymeric vesicles would be more resistant to heat exposure as polymer membranes are believed to be more robust than those formed by lipids alone. Polymersomes with a large membrane thickness (PEE_37_-PEO_40_ and PBd_46_-PEO_26_) have previously been shown to have both encapsulation and colloidal stability to autoclave conditions, although there is a slight shift to a smaller vesicle size distribution [28]. To enhance the stability of the vesicles during heat sterilisation, it has been suggested that sugars or polyols could be used to stabilise the vesicles [52] and the drug could be encapsulated after vesicles have been autoclaved [38].

Lyophilisation also caused complete contents release and loss of colloidal stability in both pure lipid and pure PBd-*b*-PEO polymer vesicles, indicating rupture and aggregation of the vesicles. The initial freezing step could cause damage to vesicles from piercing by the ice crystals that form and expansion of the encapsulated aqueous volume during freezing. However, we later show that polymer-rich vesicle compositions in particular are comparatively stable under freeze-thaw action. Therefore, damage caused by freezing is not solely responsible for the chronic impact of lyophilisation on these vesicles.

During the second step of lyophilisation, water is removed by sublimation. As water is fundamentally essential to the self-assembly of amphiphiles into vesicle polymorphs, and the degree of hydration is known to facilitate phase transitions in lyotropic assemblies, it is perhaps not surprising that this dehydration step can cause severe structural instabilities. Here, we considered whether the enhanced stability of block copolymers alone might be enough to increase the stability of vesicles to lyophilisation; however, there is no evidence for this under the conditions currently tested. However, the damaging effects of lyophilisation have been minimised for liposomes by addition of cryoprotectants to the continuous aqueous phase. Small molecule cryoprotectants, such as glycerol or trehalose and other carbohydrates, can significantly reduce aggregation, fusion and leakage. Cryoprotectants have been successfully used to enhance the stability of liposomes to lyophilisation and rehydration [43]. However, this is notoriously challenging [44], and the potential for synergistic effects from cryoprotectants in the solution phase and block copolymers blended within hybrid vesicle membranes is a worthwhile avenue for future investigation.

While autoclaving and lyophilisation would need further investigation of added molecular protectants in the solvent environment to make them viable for hybrid vesicle processing, filtration and freeze-thaw processes appeared to be more immediately viable for sterilisation and preservation.

Vesicle filtration (200 nm pore size) resulted in relatively low contents release, particularly for vesicles formed by extrusion through a 100 nm membrane. These vesicles also showed good colloidal stability. However, this is perhaps relatively unsurprising since the DLS size distribution suggests that only a small proportion of vesicles are larger than the filter pore size, although hydrodynamic sheer stresses passing through a filter pore might cause minimal destabilisation of the membrane and loss of contents.

A more stringent test of vesicle stability under filtration was undertaken with vesicles formed by extrusion through 400 nm membranes. Now, a large proportion of vesicles are larger than the 200 nm filter pore size. More significant contents release is now observed (up to 25%) and there is some loss of colloidal stability due to vesicle break-up and aggregation. Curiously, only 50 mol% PBd-*b*-PEO hybrid vesicles maintained good colloidal stability, but this might be explained by the intrinsically smaller size distribution of these hybrid vesicles when initially formed.

When comparing between different vesicle compositions, it is interesting to note that the vesicle compositions with the highest filtration stability are perhaps initially non-intuitive. A reasonable initial hypothesis might be that vesicles with a high proportion of the larger PBd-*b*-PEO block copolymer would be more stable under filtration due to enhanced mechanical robustness from this polymer. However the converse is the case, where the worst performing vesicle composition is 100% PBd_22_-PEO_14_. We interpret this to be due to the higher bending rigidity of the thicker membranes of these vesicles, offering higher resistance to deformations required to pass through the filter membrane. This is qualitatively manifested in the higher pressures that are required to force these vesicle samples across the filter membrane. These higher pressures likely have a more severe impact on encapsulation stability. Conversely, the smaller PBd_12_-PEO_11_ polymer does not have a significant impact on encapsulation stability, likely due to a similarly low bending rigidity to pure liposomes. These hybrid vesicles formed using this smaller polymer may also have enhanced membrane elasticity, imparting improved toughness to these vesicles, but this is not a statistically significant observation, likely due to the inherently low contents release that is already seen for the POPC liposomes.

Polymer-rich vesicles can also withstand several FTV cycles, making this a more viable alternative to lyophilisation for sample preservation. Lipid-rich vesicles have poor encapsulation stability under freeze-thaw action, but high polymer content likely enhances stability due to the increased stretching elasticity and higher lysis strain of polymersome membranes that is critical to their enhanced material toughness and durability. Now, membranes rich in the larger PBd_22_-PEO_14_ polymers present the best stability profile across several FTV cycles, having greater capacity to resist damage from solvent expansion stresses during freezing and the growth of ice crystals.

In summary, this initial investigation indicates that filtration and freeze-thaw are the most viable routes to sterilisation and preservation of hybrid vesicles while retaining good encapsulation and colloidal stability of these samples. A complex interplay between the bending rigidity, stretching elasticity and lysis strain of the membrane formulation determines which vesicles are most stable under each of these processes. While functional performance in the desired vesicle application will primarily dictate the optimisation of the membrane composition of vesicles, enhancing essential processing stability is a secondary consideration that cannot be completely ignored. While filtration is sufficient to sterilise small nano-vesicle samples, formulations that use larger vesicle sizes are less stable to this process and further efforts to develop autoclaving as a stable process for hybrid vesicles are worthwhile. Similarly, lyophilised vesicles would be favourable for frozen liquid samples for the preservation of vesicles for storage and transport to reduce economic costs and environmental impact. Therefore, further efforts to enhance hybrid vesicle stability to these harsher processes are still of significant interest. Furthermore, additional sterilisation processes might be considered, such as ultra-high temperature (UHT) and high temperature short time (HTST) [53] processing.

## Supplementary Materials

The following are available online at https://www.mdpi.com/2073-4360/12/4/914/s1, Table S1: DLS fitting data of PBd_12_-PEO_11_ hybrid vesicles before five filtration cycles for Figure 3c, Table S2: DLS fitting data of PBd_22_-PEO_14_ hybrid vesicles before five filtration cycles for Figure 3c, Table S3: DLS fitting data of PBd_12_-PEO_11_ hybrid vesicles after five filtration cycles for Figure 3c, Table S4: DLS fitting data of PBd_22_-PEO_14_ hybrid vesicles after five filtration cycles for Figure 3c, Table S5: DLS fitting data of PBd_12_-PEO_11_ hybrid vesicles before five filtration cycles for Figure 4c, Table S6: DLS fitting data of PBd_22_-PEO_14_ hybrid vesicles before five filtration cycles for Figure 4c, Table S7: DLS fitting data of PBd_12_-PEO_11_ hybrid vesicles after five filtration cycles for Figure 4c, Table S8: DLS fitting data of PBd_22_-PEO_14_ hybrid vesicles after five filtration cycles for Figure 4c, Table S9: DLS fitting data of PBd_12_-PEO_11_ hybrid vesicles before four FTV cycles for Figure 6c, Table S10: DLS fitting data of PBd_22_-PEO_14_ hybrid vesicles after four FTV cycles for Figure 6c, Table S11: DLS fitting data of PBd_12_-PEO_11_ hybrid vesicles after four FTV cycles for Figure 6c, Table S12: DLS fitting data of PBd_22_-PEO_14_ hybrid vesicles after four FTV cycles for Figure 6c.

## References

[1] Chemin M., Brun P.M., Lecommandoux S., Sandre O., Le Meins J.F. (2012). Hybrid polymer/lipid vesicles: Fine control of the lipid and polymer distribution in the binary membrane. Soft Matter.

[2] Chen D., Santore M.M. (2015). Hybrid copolymer-phospholipid vesicles: Phase separation resembling mixed phospholipid lamellae, but with mechanical stability and control. Soft Matter.

[3] Dao T.P.T., Fernandes F., Ibarboure E., Ferji K., Prieto M., Sandre O., Le Meins J.F. (2017). Modulation of phase separation at the micron scale and nanoscale in giant polymer/lipid hybrid unilamellar vesicles (GHUVs). Soft Matter.

[4] Henderson I.M., Paxton W.F. (2014). Salt, Shake, Fuse-Giant Hybrid Polymer/Lipid Vesicles through Mechanically Activated Fusion. Angew. Chem. Int. Ed..

[5] Hu S.W., Huang C.Y., Tsao H.K., Sheng Y.J. (2019). Hybrid membranes of lipids and diblock copolymers: From homogeneity to rafts to phase separation. Phys. Rev. E.

[6] Lim S.K., de Hoog H.P., Parikh A.N., Nallani M., Liedberg B. (2013). Hybrid, Nanoscale Phospholipid/Block Copolymer Vesicles. Polymers.

[7] Magnani C., Montis C., Mangiapia G., Mingotaud A.F., Mingotaud C., Roux C., Joseph P., Berti D., Lonettia B. (2018). Hybrid vesicles from lipids and block copolymers: Phase behavior from the micro- to the nano-scale. Colloids Surf. B Biointerfaces.

[8] Nam J., Vanderlick T.K., Beales P.A. (2012). Formation and dissolution of phospholipid domains with varying textures in hybrid lipo-polymersomes. Soft Matter.

[9] Schulz M., Binder W.H. (2015). Mixed Hybrid Lipid/Polymer Vesicles as a Novel Membrane Platform. Macromol. Rapid Commun..

[10] Schulz M., Glatte D., Meister A., Scholtysek P., Kerth A., Blume A., Bacia K., Binder W.H. (2011). Hybrid lipid/polymer giant unilamellar vesicles: Effects of incorporated biocompatible PIB-PEO block copolymers on vesicle properties. Soft Matter.

[11] Abraham T., Mao M., Tan C.E.M. (2018). Engineering approaches of smart, bio-inspired vesicles for biomedical applications. Phys. Biol..

[12] Bixner O., Bello G., Virk M., Kurzhals S., Scheberl A., Gal N., Matysik A., Kraut R., Reimhult E. (2016). Magneto-Thermal Release from Nanoscale Unilamellar Hybrid Vesicles. Chemnanomat.

[13] Cheng Z.L., Elias D.R., Kamat N.P., Johnston E.D., Poloukhtine A., Popik V., Hammer D.A., Tsourkas A. (2011). Improved Tumor Targeting of Polymer-Based Nanovesicles Using Polymer-Lipid Blends. Bioconj. Chem..

[14] Khan S., McCabe J., Hill K., Beales P.A. (2020). Biodegradable hybrid block copolymer—Lipid vesicles as potential drug delivery systems. J. Colloid Interface Sci..

[15] Mohammadi M., Taghavi S., Abnous K., Taghdisi S.M., Ramezani M., Alibolandi M. (2018). Hybrid Vesicular Drug Delivery Systems for Cancer Therapeutics. Adv. Funct. Mater..

[16] Palivan C.G., Goers R., Najer A., Zhang X.Y., Car A., Meier W. (2016). Bioinspired polymer vesicles and membranes for biological and medical applications. Chem. Soc. Rev..

[17] Panneerselvam K., Lynge M.E., Riber C.F., Mena-Hernando S., Smith A.A.A., Goldie K.N., Zelikin A.N., Stadler B. (2015). Phospholipid-polymer amphiphile hybrid assemblies and their interaction with macrophages. Biomicrofluidics.

[18] Patil S.S., Kumbhar D.D., Manwar J.V., Jadhao R.G., Bakal R.L., Wakode S. (2019). Ultrasound-Assisted Facile Synthesis of Nanostructured Hybrid Vesicle for the Nasal Delivery of Indomethacin: Response Surface Optimization, Microstructure, Stability. AAPS PharmSciTech.

[19] Pippa N., Kaditi E., Pispas S., Demetzos C. (2013). PEO-b-PCL-DPPC chimeric nanocarriers: Self-assembly aspects in aqueous and biological media and drug incorporation. Soft Matter.

[20] Pippa N., Merkouraki M., Pispas S., Demetzos C. (2013). DPPC: MPOx chimeric advanced Drug Delivery nano Systems (chi-aDDnSs): Physicochemical and structural characterization, stability and drug release studies. Int. J. Pharm..

[21] Robertson J.D., Yealland G., Avila-Olias M., Chierico L., Bandmann O., Renshaw S.A., Battaglia G. (2014). pH-Sensitive Tubular Polymersomes: Formation and Applications in Cellular Delivery. ACS Nano.

[22] Rideau E., Dimova R., Schwille P., Wurm F.R., Landfester K. (2018). Liposomes and polymersomes: A comparative review towards cell mimicking. Chem. Soc. Rev..

[23] Beales P.A., Khan S., Muench S.P., Jeuken L.J.C. (2017). Durable vesicles for reconstitution of membrane proteins in biotechnology. Biochem. Soc. Trans..

[24] Khan S., Li M.Q., Muench S.P., Jeuken L.J.C., Beales P.A. (2016). Durable proteo-hybrid vesicles for the extended functional lifetime of membrane proteins in bionanotechnology. Chem. Commun..

[25] Paxton W.F., McAninch P.T., Achyuthan K.E., Shin S.H.R., Monteith H.L. (2017). Monitoring and modulating ion traffic in hybrid lipid/polymer vesicles. Colloids Surf. B Biointerfaces.

[26] Seneviratne R., Khan S., Moscrop E., Rappolt M., Muench S.P., Jeuken L.J.C., Beales P.A. (2018). A reconstitution method for integral membrane proteins in hybrid lipid-polymer vesicles for enhanced functional durability. Methods.

[27] Jacobs M.L., Boyd M.A., Kamat N.P. (2019). Diblock copolymers enhance folding of a mechanosensitive membrane protein during cell-free expression. Proc. Natl. Acad. Sci. USA.

[28] Lee J.C.M., Bermudez H., Discher B.M., Sheehan M.A., Won Y.-Y., Bates F.S., Discher D.E. (2001). Preparation, stability, and in vitro performance of vesicles made with diblock copolymer. Biotechnol. Bioeng..

[29] Nam J., Beales P.A., Vanderlick T.K. (2011). Giant Phospholipid/Block Copolymer Hybrid Vesicles: Mixing Behavior and Domain Formation. Langmuir.

[30] Garni M., Wehr R., Avsar S.Y., John C., Palivan C., Meier W. (2019). Polymer membranes as templates for bio-applications ranging from artificial cells to active surfaces. Eur. Polym. J..

[31] Dao T.P.T., Brulet A., Fernandes F., Er-Rafik M., Ferji K., Schweins R., Chapel J.P., Schmutz F.M., Prieto M., Sandre O. (2017). Mixing Block Copolymers with Phospholipids at the Nanoscale: From Hybrid Polymer/Lipid Wormlike Micelles to Vesicles Presenting Lipid Nanodomains. Langmuir.

[32] Stoenescu R., Graff A., Meier W. (2004). Asymmetric ABC-triblock copolymer membranes induce a directed insertion of membrane proteins. Macromol. Biosci..

[33] Otrin L., Marusic N., Bednarz C., Vidakovic-Koch T., Lieberwirt I., Landfester K., Sundmacher K. (2017). Toward Artificial Mitochondrion: Mimicking Oxidative Phosphorylation in Polymer and Hybrid Membranes. Nano Lett..

[34] Du J.Z., O’Reilly R.K. (2009). Advances and challenges in smart and functional polymer vesicles. Soft Matter.

[35] Le Meins J.F., Schatz C., Lecommandoux S., Sandre O. (2013). Hybrid polymer/lipid vesicles: State of the art and future perspectives. Mater. Today.

[36] Armenante P.M., Kirpekar A.K. (1997). Sterilization in the Pharmaceutical and Biotechnology Industry. Handbook of Downstream Processing.

[37] World Health Organisation (2019). Methods of Sterilisation. The International Pharmacopoeia.

[38] Zuidam N.J., Lee S.S.L., Crommelin D.J.A. (1993). Sterilization of Liposomes by Heat-Treatment. Pharm. Res..

[39] Jang T.H., Park S.C., Yang J.H., Kim J.Y., Seok J.H., Park U.S., Choi C.W., Lee S.R., Han J. (2017). Cryopreservation and its clinical applications. Integr. Med. Res..

[40] Cuhadar S., Koseoglu M., Atay A., Dirican A. (2013). The effect of storage time and freeze-thaw cycles on the stability of serum samples. Biochem. Med..

[41] Sykes C. (2018). Time- and Temperature-Controlled Transport: Supply Chain Challenges and Solutions. Pharm. Ther..

[42] de Castro M.D., Garcia J.L. (2002). Analytical freeze-drying. Tech. Instrum. Anal. Chem..

[43] Khan I., Elhissi A., Shah M., Alhnan M., Ahmed W. (2013). Liposome-Based Carrier Systems and Devices Used for Pulmonary Drug Delivery. Biomaterials and Medical Tribology.

[44] Franzé S., Selmin F., Samaritani E., Minghetti P., Cilurzo F. (2018). Lyophilization of Liposomal Formulations: Still Necessary, Still Challenging. Pharmaceutics.

[45] Meng F., Engbers G., Feijen J. (2005). Biodegradable polymersomes as a basis for artificial cells: Encapsulation, release and targeting. J. Control. Release.

[46] Goldbach P., Brochart H., Wehrlé P., Stamm A. (1995). Sterile filtration of liposomes: Retention of encapsulated carboxyfluorescein. Int. J. Pharm..

[47] Drulyte I., Johnson R.M., Hesketh E.L., Hurdiss D.L., Scarff C.A., Porav S.A., Ranson N.A., Muench S.P., Thompson R.F. (2018). Approaches to altering particle distributions in cryo-electron microscopy sample preparation. Acta Crystallogr. Sect. D Struct. Biol..

[48] Milne J.L.S., Borgnia M.J., Bartesaghi A., Tran E.E.H., Earl L.A., Schauder D.M., Lengyel J., Pierson J., Patwardhan A., Subramaniam S. (2013). Cryo-electron microscopy—A primer for the non-microscopist. FEBS J..

[49] Rice W.J., Cheng A., Noble A.J., Eng E.T., Kim L.Y., Carragher B., Potter C.S. (2018). Routine determination of ice thickness for cryo-EM grids. J. Struct. Biol..

[50] Sejwal K., Chami M., Baumgartner P., Kowal J., Mller S.A., Stahlberg H. (2017). Proteoliposomes—A system to study membrane proteins under buffer gradients by cryo-EM. Nanotechnol. Rev..

[51] Karlsson G. (2001). Thickness measurements of lacey carbon films. J. Microsc. Oxf..

[52] Kikuchi H., Carlsson A., Yachi K., Hirota S. (1991). Possibility of heat sterilization of liposomes. Chem. Pharm. Bull..

[53] Mann A., Kiefer M., Leuenberger H. (2001). Thermal sterilization of heat-sensitive products using high-temperature short-time sterilization. J. Pharm. Sci..

