# Supplementary material for: Hybrid Vesicle Stability under Sterilisation and Preservation Processes Used in the Manufacture of Medicinal Formulations
Source: Polymers (Basel). 2020 Apr 15;12(4):914. doi: 10.3390/polym12040914 (PMC7240416; doi:10.3390/polym12040914)
Supplement: Supplementary file 1 [file polymers-12-00914-s001.pdf]

Hybrid Vesicle Stability under Sterilisation and Preservation Processes Used in the Manufacture of Medicinal Formulations

Rashmi Seneviratne, Lars J.C. Jeuken, Michael Rappolt and Paul A. Beales

Table S1. DLS fitting data of PBd_{12}-PEO_{11} hybrid vesicles before five filtration cycles for Figure 3c.

PBd_{12}-PEO_{11} (mol%)	Z-average (d.nm)		Average Intensity Diameter (d.nm)		PDI	
	Average	Standard Deviation	Average	Standard Deviation	Average	Standard Deviation
0	123.2	120.0	131.9	7.7	0.096	0.018
25	107.8	0.1	122.5	0.6	0.120	0.011
50	95.7	4.3	115.2	4.6	0.163	0.014
75	90.4	1.8	110.6	1.8	0.174	0.003
100	99.8	3.8	122.7	6.8	0.183	0.015

Table S2. DLS fitting data of PBd_{22}-PEO_{14} hybrid vesicles before five filtration cycles for Figure 3c.

PBd_{22}-PEO_{14} (mol%)	Z-average (d.nm)		Average Intensity Diameter (d.nm)		PDI	
	Average	Standard Deviation	Average	Standard Deviation	Average	Standard Deviation
0	123.2	120.0	131.9	7.7	0.096	0.018
25	97.2	1.5	114.5	6.0	0.154	0.003
50	108.0	2.1	138.9	2.4	0.213	0.022
75	114.8	5.4	135.3	5.8	0.145	0.018
100	160.8	17.0	189.0	21.1	0.16	0.044

Table S3. DLS fitting data of PBd_{12}-PEO_{11} hybrid vesicles after five filtration cycles for Figure 3c.

PBd_{12}-PEO_{11} (mol%)	Z-average (d.nm)		Average Intensity Diameter (d.nm)		PDI	
	Average	Standard Deviation	Average	Standard Deviation	Average	Standard Deviation
0	121.8	9.3	132.0	7.8	0.085	0.024
25	107.6	1.7	126.3	5.3	0.134	0.002
50	96.1	5.3	1595.4	2643.2	0.180	0.017
75	124.0	57.7	287.8	306.4	0.218	0.083
100	99.6	1.2	127.4	0.7	0.204	0.011

Table S4. DLS fitting data of PBd_{22}-PEO_{14} hybrid vesicles after five filtration cycles for Figure 3c.

PBd_{22}-PEO_{14} (mol%)	Z-average (d.nm)		Average Intensity Diameter (d.nm)		PDI	
	Average	Standard Deviation	Average	Standard Deviation	Average	Standard Deviation
0	121.8	9.3	132.0	7.8	0.085	0.024
25	96.1	0.8	111.2	2.5	0.147	0.013
50	105.9	2.0	132.4	1.0	0.185	0.015
75	116.8	4.8	137.2	5.9	0.137	0.010
100	149.1	1.8	176.0	3.3	0.145	0.011

Table S5. DLS fitting data of PBd_{12}-PEO_{11} hybrid vesicles before five filtration cycles for Figure 4c.

PBd_{12}-PEO_{11} (mol%)	Z-average (d.nm)		Average Intensity Diameter (d.nm)		PDI	
	Average	Standard Deviation	Average	Standard Deviation	Average	Standard Deviation
0	318.8	36.5	263.5	34.5	0.417	0.050
50	106.9	1.0	1588.6	850.9	0.247	0.002
100	160.6	68.5	2448.7	1354.4	0.221	0.135

Table S6. DLS fitting data of PBd_{22}-PEO_{14} hybrid vesicles before five filtration cycles for Figure 4c.

PBd_{22}-PEO_{14} (mol%)	Z-average (d.nm)		Average Intensity Diameter (d.nm)		PDI	
	Average	Standard Deviation	Average	Standard Deviation	Average	Standard Deviation
0	318.8	36.5	263.5	34.5	0.417	0.050
50	123.7	0.9	182.2	8.5	0.279	0.014
100	261.4	87.4	1447.4	948.3	0.491	0.055

Table S7. DLS fitting data of PBd_{12}-PEO_{11} hybrid vesicles after five filtration cycles for Figure 4c.

PBd_{12}-PEO_{11} (mol%)	Z-average (d.nm)		Average Intensity Diameter (d.nm)		PDI	
	Average	Standard Deviation	Average	Standard Deviation	Average	Standard Deviation
0	822.9	856.1	719.9	555.9	0.493	0.444
50	94.4	8.3	120.1	35.0	0.211	0.004
100	227.6	169.4	2291.5	1123.4	0.447	0.417

Table S8. DLS fitting data of PBd_{22}-PEO_{14} hybrid vesicles after five filtration cycles for Figure 4c.

PBd_{22}-PEO_{14} (mol%)	Z-average (d.nm)		Average Intensity Diameter (d.nm)		PDI	
	Average	Standard Deviation	Average	Standard Deviation	Average	Standard Deviation
0	822.9	856.1	719.9	555.9	0.493	0.444
50	119.8	30.2	164.9	47.9	0.269	0.029
100	579.4	707.4	798.75	699.9	0.524	0.225

Table S9. DLS fitting data of PBd_{12}-PEO_{11} hybrid vesicles before four FTV cycles for Figure 6c.

PBd_{12}-PEO_{11} (mol%)	Z-average (d.nm)		Average Intensity Diameter (d.nm)		PDI	
	Average	Standard Deviation	Average	Standard Deviation	Average	Standard Deviation
0	123.2	120.0	131.9	7.7	0.096	0.018
25	107.8	0.1	122.5	0.6	0.120	0.011
50	95.7	4.3	115.2	4.6	0.163	0.014
75	90.4	1.8	110.6	1.8	0.174	0.003
100	99.8	3.8	122.7	6.8	0.183	0.015

Table S10. DLS fitting data of PBd22-PEO14 hybrid vesicles after four FTV cycles for Figure 6c.

PBd22-PEO14 (mol%)	Z-average (d.nm)		Average Intensity Diameter (d.nm)		PDI	
	Average	Standard Deviation	Average	Standard Deviation	Average	Standard Deviation
0	123.2	120.0	131.9	7.7	0.096	0.018
25	97.2	1.5	114.5	6.0	0.154	0.003
50	108.0	2.1	138.9	2.4	0.213	0.022
75	114.8	5.4	135.3	5.8	0.145	0.018
100	160.8	17.0	189.0	21.1	0.16	0.044

Table S11. DLS fitting data of PBd12-PEO11 hybrid vesicles after four FTV cycles for Figure 6c.

PBd12-PEO11 (mol%)	Z-average (d.nm)		Average Intensity Diameter (d.nm)		PDI	
	Average	Standard Deviation	Average	Standard Deviation	Average	Standard Deviation
0	116.8	19.5	136.2	32.9	0.135	0.074
25	82.3	5.3	93.5	2.5	0.168	0.009
50	81.7	2.0	100.5	2.3	0.178	0.007
75	80.6	2.0	98.4	0.9	0.172	0.009
100	90.9	1.6	109.6	7.4	0.177	0.015

Table S12. DLS fitting data of PBd22-PEO14 hybrid vesicles after four FTV cycles for Figure 6c.

PBd22-PEO14 (mol%)	Z-average (d.nm)		Average Intensity Diameter (d.nm)		PDI	
	Average	Standard Deviation	Average	Standard Deviation	Average	Standard Deviation
0	116.8	19.5	136.2	32.9	0.135	0.074
25	379.3	511.2	863.8	886.8	0.26	0.119
50	101.0	1.4	125.4	1.1	0.191	0.016
75	157.2	75.0	313.5	312.5	0.260	0.210
100	154.2	4.8	181.4	8.0	0.139	0.015
